# Adapting the visuo-haptic perception through muscle coactivation

**DOI:** 10.1038/s41598-021-01344-w

**Published:** 2021-11-09

**Authors:** Gerolamo Carboni, Thrishantha Nanayakkara, Atsushi Takagi, Etienne Burdet

**Affiliations:** 1 Imperial College of Science, Technology and Medicine, SW7 2AZ London, UK; 2 NTT Communication Science Laboratories, 3-1 Morinosato Wakamiya, Atsugi, Kanagawa 243-0198 Japan

**Keywords:** Biophysical models, Human behaviour

## Abstract

While the nervous system can coordinate muscles’ activation to shape the mechanical interaction with the environment, it is unclear if and how the arm’s coactivation influences visuo-haptic perception and motion planning. Here we show that the nervous system can voluntarily coactivate muscles to improve the quality of the haptic percept. Subjects tracked a randomly moving visual target they were physically coupled to through a virtual elastic band, where the stiffness of the coupling increased with wrist coactivation. Subjects initially relied on vision alone to track the target, but with practice they learned to combine the visual and haptic percepts in a Bayesian manner to improve their tracking performance. This improvement cannot be explained by the stronger mechanical guidance from the elastic band. These results suggest that with practice the nervous system can learn to integrate a novel haptic percept with vision in an optimal fashion.

## Introduction

Executing actions with the hands relies on the nervous system (NS) integrating various sensory modalities. Of particular interest for this study is how the NS incorporates visual and haptic information, where haptics describes the synthesis of skin and muscle sensing. In order to generate appropriate motor commands, the NS must also consider the arm muscle mechanics. Muscles are viscoelastic actuators that tend to shorten and stiffen with activation^1^. It is known that the NS can coordinate muscles’ activation to shape the mechanical interaction with the environment^2,3^. However, it is unclear how this affects visuo-haptic perception^4^. Could the NS adapt the limb’s viscoelasticity to improve sensing and plan motion correspondingly?

Examples of such adaptive sensing in human sensorimotor control include the pupil’s dilation to improve visual sensitivity and the influence of $\gamma$-motorneuron’s on muscle spindle sensitivity to optimise proprioception^5^. In both of these cases sensing can be studied independently from motor actions. In contrast, haptic sensing, synthesizing information from sensors in the skin, muscles and tendons during contact with the environment, cannot be separated from the mechanical interaction. This makes it challenging to investigate the haptic percept and dissociate it from the mechanical effect of adapting the limb’s viscoelasticity.

**Figure 1:**
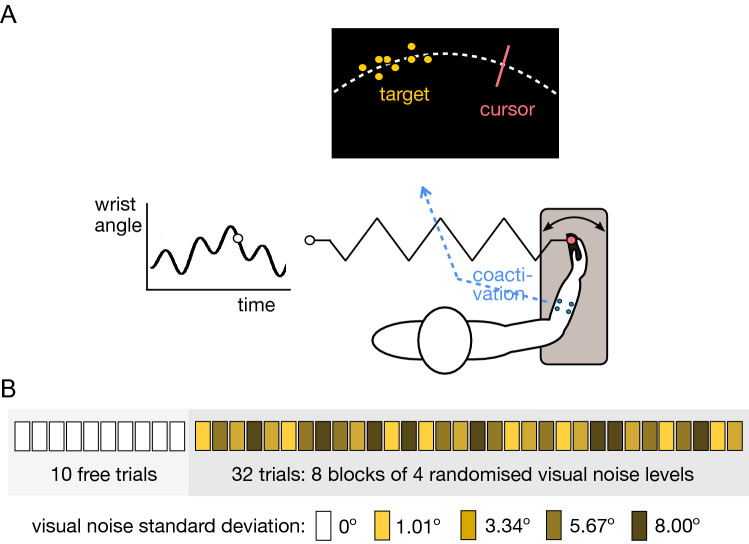
Schematic of the experimental task and protocol. (**A**) Subjects tracked a randomly moving target with their wrist flexion-extension movement while being connected to the target trajectory through a virtual spring. The spring’s stiffness increased with the coactivation of a flexor–extensor muscle pair, measured from their normalized myoelectrical activity. (**B**) The experimental protocol consisted of 10 training trials and 32 visual noise trials. The virtual spring was implemented in the visual noise trials.

To investigate whether the NS can actively control the viscoelasticity of a limb to improve the haptic perception, we observed how subjects used wrist flexion and extension to track a moving target that they were physically coupled to by a virtual elastic band. The stiffness of the virtual coupling was proportionally increased with the *cocontraction* of the wrist antagonistic muscle pair (Fig. 1A). The interaction torque exerted by the robotic interface on the connected wrist was (in Nm)

1 $$\begin{aligned} \tau (t) = \frac{u(t)}{8} \, [q^*\,(t) - q(t)], \end{aligned}$$

where the cocontraction *u*(*t*) in Nm/° was estimated as described in the Methods. The target movement was the multisine function

2 $$\begin{aligned} q^*\,(t) \equiv \,\, 18.5^{\circ } \, \sin \, \left( \, \frac{\pi t}{1.547} \, \right) \, \sin \,\left( \,\frac{\pi t}{2.875}\,\right) , \quad 0\le t\le 20\,s. \end{aligned}$$

In order to study the visuo-haptic perception, different amounts of visual noise were imposed on the target on select trials (Fig. 1B). If subjects tried to improve the haptic percept by increasing the wrist’s viscoelasticity, cocontraction should be greater in trials with higher visual noise. We tested this hypothesis by analyzing the evolution of cocontraction and the *tracking error*

3 $$\begin{aligned} e \equiv \, \left( \frac{1}{T} \, \int _0^T \left[ q^*\,(t) - q(t) \right] ^2 dt \right) ^\frac{1}{2}, \quad T \equiv 20\,s. \end{aligned}$$

Finally, we compared four different models of visuo-haptic perception to test which combination of visual and haptic information could best explain the task performance.

## Results

**Figure 2:**
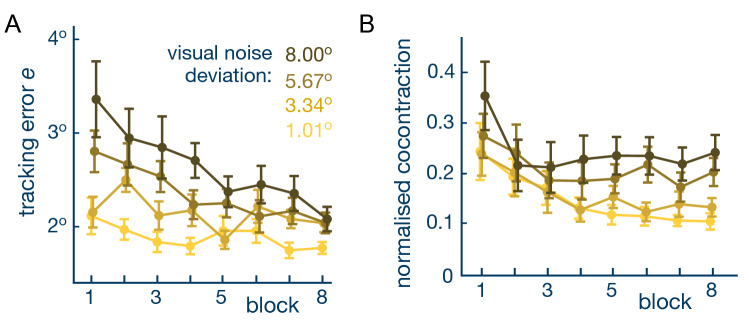
Tracking error and muscle cocontraction adapted with practice. The figures shows the mean values with standard error bar over all trials of each subject in the corresponding block. (**A**) The population mean tracking error and the associated variability decreased with trials for every level of visual noise. (**B**) The normalised cocontraction was initially large in the first block of trials for all visual noise conditions and decreased with practise. The level to which the cocontraction converged increases with the visual noise imposed on the target.

Figure 2 shows the square root of square error to the target over a trial or tracking error and the mean normalised coactivation (Eq. 12) as a function of the block number, separately for each level of visual noise. It can be seen in Fig. 2A that the tracking error was large, but gradually decreased over blocks. The effect of training was measured by comparing the error in the first and last blocks. A two-way repeated measures ANOVA showed that both the visual noise ($\hbox {p}<0.001$, $\hbox {F}(3,40)=8.67$) and the training effect ($\hbox {p}=0.002$, $\hbox {F}(1,12)=14.34$) significantly affected the error. Post-hoc comparisons using Tukey’s honestly significant difference (HSD) test confirmed a decrease of tracking error with training for the two higher levels of visual noise ($\hbox {p}=0.027$ and $\hbox {p}<0.001$ respectively) with an overall comparable performance for all visual noise levels in the last block ($\hbox {p}>0.86$).

To understand how muscle activation depends on the level of visual noise and training, we then looked at the mean cocontraction measured over a whole trial. Note that the noise was designed so that subjects could not filter the cloudy target easily by guessing its middle (as can be seen on the *video* included as supplementary material). The cocontraction was normalized to allow comparison between different participants. A two-way repeated measures ANOVA revealed a significant effect of both visual noise level ($\hbox {p}<0.001, \hbox {F}(3,40)=8.3$) and training ($\hbox {p}=0.01, \hbox {F}(1,12)=8.9$) on the normalized cocontraction. Post-hoc comparisons confirmed that the normalized cocontraction in the first block was comparable across all visual noise levels, but different between the lowest and highest visual noise levels in the last block ($\hbox {p}=0.02$). This result came as consequence of a decrease in activation between pre and post training trial ($\hbox {p}<0.020$ for $\sigma _v = 1.01^\circ$) so that the normalized cocontraction decreased by a larger margin when the visual noise was lower. This yielded a normalized cocontraction in the final three blocks that increased monotonically with the level of visual noise ($\hbox {slope}=0.0185\pm 0.002$, one-sample t-test $\hbox {t}(2)=13.15$, $\hbox {p}<0.006$). This suggests that the NS adapts the body’s stiffness to modify the haptic guidance in response to visual noise on the target.

The participants improved their performance with training, and also modified the amount of cocontraction depending on the level of visual noise imposed on the target. While learning is known to be generally accompanied by a decrease of muscle cocontraction^6–8^, this would not explain the distinct cocontraction levels observed at the end of the learning phase for the different visual noise conditions. Why did subjects increase cocontraction as a function of visual noise? To address this question, we analyzed how the sensory information of the target’s motion from vision and haptics was combined by the NS. We considered the tracking performance as a linear function of the amount of visual error $e_v$ and haptic error $e_h$:

4 $$\begin{aligned} e = \alpha \,e_v + \beta \,e_h, \quad \alpha , \beta >0. \end{aligned}$$

**Figure 3:**
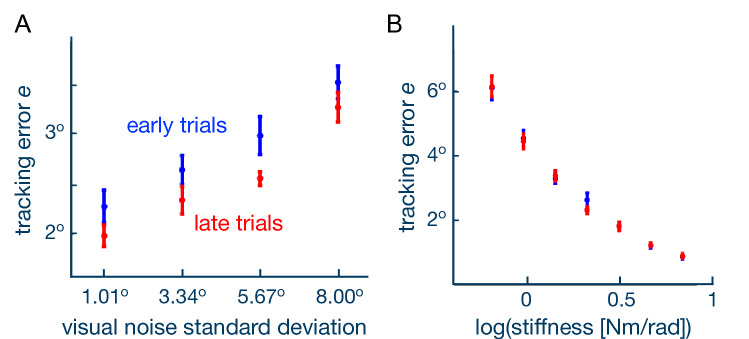
Two control experiments were carried out to assess the influence of visual noise and connection stiffness on the tracking error. (**A**) In the control experiment with visual noise the tracking error linearly grows with visual noise, and decreases across all noise levels with practise. (**B**) In the experiment with haptic feedback only, the tracking error decreases with the log of the connection stiffness (validated with linear mixed-effect analysis: $\hbox {p}<1\hbox {e}{-}09$). This relationship does not change with practice.

A *visual control experiment* was first carried out to evaluate the influence of the visual noise on the tracking performance without elastic force (as detailed in the Methods). The tracking error was positively, linearly correlated with the visual noise imposed on the target (Fig. 3A,^9^) and decreased with learning, yielding consistent performance improvement among the visual noise conditions ($\hbox {p}=6.60$e−05 for initial-last trials, paired t-test). This was modelled as

5 $$\begin{aligned} e_v(\sigma _v) = \alpha _{v} + \beta _{v} \, \sigma _v, \end{aligned}$$

where $\alpha _{v}=-11.67, \beta _{v}=0.13$ were identified from a least-squares linear regression with data from the last two trials.

Next, a *haptic experiment* was performed to measure how the tracking error depended on the elasticity of the virtual band. In this control experiment (detailed in the Methods), no visual feedback was provided while the wrist was connected to the target reference trajectory with seven selected levels of elasticity, $\kappa$, through

6 $$\begin{aligned} \tau (t) = \kappa \, [q^*(t) - q(t)]. \end{aligned}$$

As expected, the tracking error decreased with increasing elasticity (Fig. 3B), which was modelled as a quadratic function

7 $$\begin{aligned} e_h(\kappa ) = \alpha _h \,+ \beta _h \, log_{10}(\kappa ) + \, \gamma _h \,[\log _{10}(\kappa )]^2 \end{aligned}$$

where $\alpha _h= 4.42$, $\beta _h=-7.59$, $\gamma _h=4.15$ were identified from a least-square fit. This relation between tracking error and coupling stiffness did not change with learning. In order to weight the distinct contributions from vision and haptics on the tracking performance, we need to calculate the standard deviation due to the connection compliance, similar to the visual noise. This is done by equalizing the error function due to visual noise Eq. (5) with the tracking error due to compliance Eq. (7): $e(\kappa ) \equiv e(\sigma _v)$, yielding

8 $$\begin{aligned} \sigma _{h_{ij}} = \frac{\alpha _h - \alpha _v + \beta _h \, \log _{10}(\kappa _{ij}) + \, \gamma _h \,{\log _{10}(\kappa _{ij})}^2}{\beta _v} \,[^\circ ] \end{aligned}$$

for *i*th subject and *j*th visual noise level.

Four models expressing different combinations of the visual and haptic information could then be tested using Eq. 4. The *visual dominance model* expresses that only information of the target’s motion from vision is used to track the target, such that the performance is fully explained by the reliability of vision alone ($\alpha =1$, $\beta =0$). The *haptic dominance model* expresses the converse, where haptic information alone is used to track the target ($\alpha =0$, $\beta =1$). In the *averaging model*, visual and haptic information are weighted equally to track the target ($\alpha =\beta =1/2$). The *Bayesian integration model* assumes that a stochastically optimal weighted average is used to minimize the prediction error considering information from vision and haptic sensing:

9 $$\begin{aligned} \alpha = \frac{\sigma _h^2}{\sigma _v^2 + \sigma _h^2},\quad \beta = \frac{\sigma _v^2}{\sigma _v^2 + \sigma _h^2} \end{aligned}$$

with $\sigma _v$ and $\sigma _h$ the standard deviations of visual and haptic noise, respectively. Each model yielded a different prediction of the task performance for a given visual noise level and cocontraction value. By comparing the predicted task error and the empirically measured error, we could assess which model best explained the sensory combination of vision and haptics.

**Figure 4:**
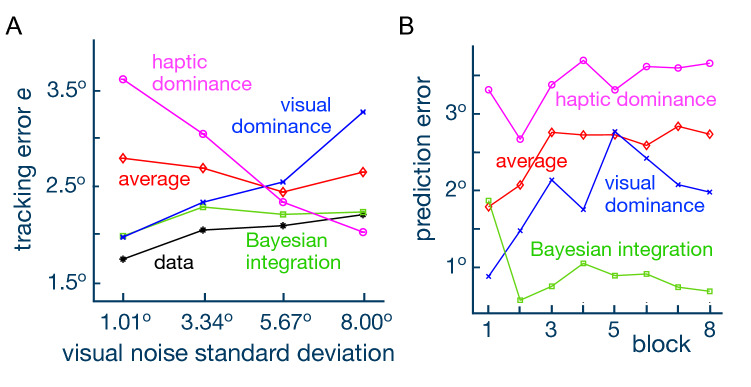
The best model that minimised the prediction error was the Bayesian integration of visual and haptic information. (**A**) The tracking performance predicted by each model is plotted as a function of the visual noise. With the increase of visual noise, the *visual dominance* model diverged from the experimental data. In contrast, the *haptic dominance* model predicted large error with low visual noise, which was different from the data. The error predicted by the *Bayesian integration* model was closest to the empirical data for all levels of visual noise. (**B**) The prediction error from each model is plotted as a function of the block number. In the first block, the *visual dominance* model yielded the lowest prediction error. From the second block onwards, the *Bayesian integration* model outperformed the others, with both the *visual dominance* and the *averaging* models’ predictions inflating with trials. The *haptic dominance* model yielded the worst prediction.

We first analyzed how each model’s predicted performance depended on the visual noise condition (Fig. 4A). Unsurprisingly, the visual dominance model predicted greater error as visual noise increased. Conversely, the haptic dominance model predicted decreasing error with visual noise since the cocontraction was greater with higher visual noise. The averaging model predicted a roughly constant error irrespective of the visual noise value. The predicted performance from the Bayesian integration model was the nearest to the empirical data. A two way repeated measure ANOVA of the difference between the model and experimental data revealed a significant influence of the different models on the estimation ($\hbox {p}<0.001, \hbox {F}(3,56)=5.75$), while the effect of visual noise level had a negligible effect. Post-hoc comparisons both confirmed the similarity of the Bayesian integration model predicted with experimental data ($\hbox {p}>0.1$), and confirmed their difference to predictions from the other models ($\hbox {p}<0.001$).

We then examined how the prediction error (computed as the maximum likelihood estimate of the difference between the task error predicted by each model and the empirical error from the experimental data) changed as a function of the trials’ blocks. The visual dominance model yielded the smallest prediction error in the first block (Fig. 4B). However, its prediction faltered in later blocks. The haptic dominance model yielded the worst prediction of all four models, with the prediction error growing in later blocks. The averaging model layed in between the visual and haptic dominance models, which indicates that the information from the two sensory channels was not weighted equally by the NS. The Bayesian integration model was the best model in all blocks but the first. This suggests that subjects may have relied mainly on vision in the first block to track the target, but gradually learned to integrate the haptic percept from the second block onwards to improve performance.

## Discussion

The field of morphological computation has analysed how animals’ biomechanical design facilitates their functions^10,11^, and human motor control has revealed how the NS controls the limbs’ viscoelasticity to shape the mechanical interaction with the environment^2,3,12^. Different from these morphological and motor adaptations, our results provide evidence that the NS actively controls the limbs’ viscoelasticity to improve perception. Our results showed that muscle coactivation was adapted to increase the accuracy of visuo-haptic perception by considering the reliability of visual and haptic information. While previous studies have provided qualitative evidence of adaptive sensing in human motor control^5,13,14^, we could quantify the performance improvement resulting from the increase in muscle cocontraction. This enabled us to identify the stochastically optimal mechanism used by the NS to regulate cocontraction and improve visuo-haptic perception.

The association of various sensory modalities and their stochastically optimal integration has been reported in numerous studies^4,15^, where the reliability of each sensory input was manipulated externally by the experimenter. In contrast, the reliability of the haptic information in our experiment was controlled by the participants themselves. Importantly, while the subjects could have coactivated their wrist indiscriminately to yield superior performance through haptic guidance to the target, they only increased coactivation to maintain the same performance level independently of the visual noise level. This points to a trade-off between task performance and effort, a common metric used to explain human motor behaviors, from reaching to interaction tasks^16–18^.

The time evolution of the prediction for each model offers some insight into the learning process. While the visual dominance model was superior in the first block, it gradually diverged in proceeding blocks, suggesting that vision was used predominantly only early on. The Bayesian integration model was best from the second block onwards, suggesting that the NS had learned to adapt the wrist’s cocontraction to sense the haptic percept relating to the target’s trajectory and integrate it with vision. This requires the NS to understand the relationship between the muscle cocontraction and the mechanical guidance to the target. At this point, it is unclear how the brain models or learns this relationship. While some recent studies have examined the role of cocontraction and its effects on motor learning^19,20^, questions remain concerning the ability of the NS to recognize sources of additional sensory information pertaining to a task, how it decides to integrate these additional sources with existing sensory channels, and how this decision-making process influences motion planning and the formation of motor memories.

## Methods

### Participants

The experiments described below were approved by the Joint Research Compliance Office at Imperial College London. Participants without known sensorimotor impairments were recruited to take part in the main experiment and in the two control experiments. 15 subjects (7 females, aged 23.46 ± 2.39 years old) carried out the main experiment. Each participant gave written informed consent prior to participation. 30/31 participants, between main and control experiments, were right-handed as assessed using the Edinburgh Handedness Inventory^21^.

### Apparatus

Each participant was seated comfortably on a height-adjustable chair next to the Hi5 robotic interface^22^, and held a handle with their dominant wrist. A screen placed in front of the participant provided visual feedback of the task with a cursor indicating the current wrist position (Fig. 1A). The Hi5 handle is connected to a current-controlled DC motor (MSS8, Mavilor) that can exert torques of up to 15 Nm, and equipped with a differential encoder (RI 58-O, Hengstler) to measure the wrist angle and a force sensor (TRT-100, Transducer Technologies) to measure the exerted torque in the range [0,11.29] Nm. The Hi5 system is controlled at 1kHz using Labview Real-Time v14.0 (National Instruments) and a data acquisition board (DAQ-PCI-6221, National Instruments), while data was recorded at 100 Hz.

The activation of two antagonist wrist muscles, the flexor carpi radialis (FCR) and extensor carpi radialis longus (ECRL), were recorded with surface electrodes using a medically certified non-invasive electromyography system (g.LADYBird + g.GAMMABox + g.BSamp, g.Tec). The raw muscle activity was high-pass filtered at 100 Hz, rectified, then low-pass filtered at 10 Hz using a second-order Butterworth filter to yield the filtered muscle activity.

### Normalized coactivation

Every experiment started with an *EMG normalization* to map the raw muscle activity (in mV) to a corresponding torque value (in Nm). The subject placed their wrist in the most comfortable neutral posture, which was set to 0$^\circ$. Constrained at that posture, they were then instructed to sequentially (*i*) flex, or extend the wrist to exert a torque, or (*ii*) maximally co-contract in order to keep the wrist position stable during a 3 Hz sinusoidal positional disturbance of 10$^\circ$ amplitude. Each phase was 4 s long with a 5 s rest period between consecutive contraction phases to avoid fatigue, which was used as a reference activity in the relaxed condition. This was repeated four times at flexion/extension torque levels {1, 2, 3, 4} Nm and $\{-1,-2,-3,-4\}$ Nm, respectively. For each subject, the recorded muscle activity was linearly regressed against the torque values to estimate the relationship between them. The torque of the flexor muscle was modelled from the filtered EMG signal $u_f$ as

10 $$\begin{aligned} \tau _f(t) = \alpha _0 \, u_f(t) \, + \, \alpha _1, \quad \alpha _0, \alpha _1 > 0, \end{aligned}$$

and similarly for the torque of the extensor muscle $\tau _e$. *Muscle cocontraction* was then computed as

11 $$\begin{aligned} u(t) \equiv \, {\min }\{\tau _f(t),\tau _e(t)\}. \end{aligned}$$

The average coactivation over all participants (as shown in Fig. 2B) was computed from each participant’s *normalised coactivation*, calculated as

12 $$\begin{aligned} u_n \equiv \frac{\overline{u} - \overline{u}_{\min }}{\overline{u}_{\max }-\overline{u}_{\min }}, \quad \overline{u} \equiv \, \frac{1}{T} \,\int _0^T u(t) \, dt, \quad T=20\,s \end{aligned}$$

where $\overline{u}_{\min }$ and $\overline{u}_{\max }$ are the minimum and maximum of the means of all trials of the specific participant.

### Main experiment

The target trajectory was displayed on the screen either as an 8 mm diameter circle or as a *cloud* of eight normally distributed dots around the target (Fig. 1A), depending on the experimental condition. The cloud of dots were defined by three parameters, randomly picked from independent Gaussian distributions: the vertical distance to the target position $\eta \in$ N(0, 15 mm), the angular distance to the target position $\eta _q \in \hbox {N}(0,\,0<\sigma _v<8.00^\circ )$, and the angular velocity $\eta _{\dot{q}} \in \hbox {N}(0,\,4^\circ /\hbox {s})$. The amplitude of visual noise was controlled by the angular distance deviation, while both the vertical and the angular velocity deviations were kept constant. The dots were updated sequentially so that each dot was replaced every 100 ms. An example of the tracking with visual noise is provided in the video at https://www.youtube.com/watch?v=N_J6mMukDa8. Informed consent has been obtained from the participant to publish the identity revealing images in the supplementary video.

To get the subject accustomed with the Hi5 interface, the experiment started with a *free phase* in which no interaction torque was exerted on the wrist and the target was not noisy. This was followed by an *interaction phase* in which the subject’s wrist position was connected to the target with an elastic force. Subjects were informed of the possibility to regulate the coupling stiffness by co-contracting or relaxing their wrist muscles, and of the transition from free trials to a phase of interaction trials. They were instructed not to resist large torques provided by the motor. Each subject was instructed to take small breaks when feeling (mental or physical) fatigue during the course of the experiment.

The *experimental protocol* consisted of 10 free trials followed by 32 interaction trials split into 8 blocks. The 4 trials of each block used a different value of $\sigma _v \in \{1.01^\circ ,3.34^\circ ,5.67^\circ ,8.00^\circ \}$ presented in a random order in each block (Fig. 1B). We assumed that the ordering of the blocks has no effect. After each 20 s long trial, the target disappeared and the participant was required to place their cursor on the starting position at the center of the screen. The next trial then started after a 5 s rest period and a 3 s countdown.

### Control experiments

Eight right-handed subjects not involved in the main experiment (25.01 ± 0.53 years old, 2 female) participated in the *visual control experiment*. The task was similar to the main experiment, consisting of an 8 trial training phase and a 32 trial testing phase, with each trial lasting 20 s. The training phase consisted of 8 trials without visual noise where the target’s position was displayed with a single point. In the testing phase the trials were organized into 8 blocks, each containing 4 trials with visual noise $\sigma _v$ $\in${1.01$^\circ$, 3.34$^\circ$, 5.67$^\circ$, 8.00$^\circ$}. The four levels of visual noise were randomized in each block.

Another eight subjects (7/8 right-handed, 26.75 ± 1.28 years old, 4 female) participated in the *haptic control experiment*. The experiment was structured in 5 blocks of 7 elasticity $\kappa \in \{0.011,0.016,0.025,0.037,0.055,0.081,0.120\}$ Nm/$^\circ$, presented in random order. In the interaction phase totalling 35 trials, the subjects also experienced an elastic force to the target.

## Supplementary information

Supplementary Information 1.

Supplementary Information 2.
