# Author Correction: Adapting the visuo-haptic perception through muscle coactivation

**DOI:** 10.1038/s41598-022-23215-8

**Published:** 2022-11-02

**Authors:** Gerolamo Carboni, Thrishantha Nanayakkara, Atsushi Takagi, Etienne Burdet

**Affiliations:** 1 Imperial College of Science, Technology and Medicine, London, SW7 2AZ UK; 2 NTT Communication Science Laboratories, 3-1 Morinosato Wakamiya, Atsugi, Kanagawa 243-0198 Japan

Correction to: *Scientific Reports* https://doi.org/10.1038/s41598-021-01344-w, published online 09 November 2021

The Funding section in the original version of this Article was omitted. The Funding section now reads:

"This work was funded in part by the EU H2020 grants COGIMON (ICT 644727) and PH-CODING (FETOPEN 829186).”

The original Article has been corrected.

