# Supplementary material for: Adapting the visuo-haptic perception through muscle coactivation
Source: Sci Rep. 2021 Nov 9;11:21986. doi: 10.1038/s41598-021-01344-w (PMC8578662; doi:10.1038/s41598-021-01344-w)
Supplement: Supplementary file 2 — Supplementary Information 2. [file 41598_2021_1344_MOESM2_ESM.docx]

Supplementary video 1: Example of tracking task with visual noise using the Hi5 robotic interface. The subject tracks a cloud of dots by moving a cursor through wrist's flexion and extension.
